# Supplementary material for: Maternal occupation and risk of adverse fetal outcomes in Tanzania: A hospital-based cross-sectional study
Source: PLoS One. 2025 Mar 18;20(3):e0319653. doi: 10.1371/journal.pone.0319653 (PMC11918367; doi:10.1371/journal.pone.0319653)
Supplement: S1 File — (DOCX) [file pone.0319653.s002.docx]

**Swahili version of the questionnaire for post-delivery women DODOSO**

Wodi_____________ Namba ya dodoso ____________

Tarehe ya Mahojiano_____________

| **SEHEMU YA 1: TAARIFA ZA AWALI** | | | |
| --- | --- | --- | --- |
| **Na.** | | **MASWALI** | **MAJIBU** |
| 1. | | Una umri gani? (*miaka*) | ……… |
| 2 | | Hali ya ndoa *(weka alama ya vema kwenye jibu husika)* | 1. Sijaolewa 2. Nimeolewa 3. Tumeachana 4. Mjane |
| 3. | | Kiwango cha elimu? *(weka alama ya vema kwenye jibu husika)* | 1. Sina elimu 2. Elimu ya msingi 3. Elimu ya sekondari 4. Elimu ya juu   (Eleza)……………… |
| 4. | | Je una kisukari? | 1. Ndio 2. Hapana |
| 5. | | Je una tatizo lolote la shinikizo la damu? | 1. Ndio 2. Hapana |
| **SEHEMU YA 2: SIFA ZA KAZI** | | | |
| **6.** | Umekuwa ukifanya kazi kabla ya kushika ujauzito? | 1. Ndio 2. Hapana | |
| **7.** | Kama ndio, ni aina gani ya kazi? | 1. Mama wa nyumbani 2. Muhudumu wa afya 3. Walinzi, polisi 4. Usafi wa barabara 5. Mama ntilie 6. Wafanyakazi wa saluni za kike 7. Wafanyakazi wa ofisini 8. Walimu | |

|  |  | 1. Wakulima 2. Kilimo cha samaki 3. Viwanda vya nguo 4. Biashara ndogondogo   Nyinginezo taja…………………  …………………………… |
| --- | --- | --- |
| **8** | Ni sifa gani ya kazi zilizotajwa hapo zipo kwenye kazi zako? (Tick where appropriate) | 1. Kusimama kwa muda mrefu 2. Kukaa kwa muda mrefu 3. Kuchuchumaa au kukaa muda mrefu. 4. Kubeba mizigo (zaidi ya   kilo 5)   1. Kusogeza mizigo mizito   (zaidi ya kilo 20)   1. Kazi zinazohitaji kupenyeza mkono kupata vitu vilivyo mbali 2. kazi zilizo na zana za kutetemesha 3. kazi zenye mazingira ya kelele 4. kufanya kazi kwenye misuli isiyofaa 5. Kufanya kazi katika nafasi sawa muda mrefu 6. Kufanya kazi za kurudiarudia muda mrefu 7. Kufanya kazi kwenye mazingira ya kemikali |

|  |  | mashambani, nyumbani |
| --- | --- | --- |
| **9.** | Je, uliendelea kufanya kazi baada ya kupata ujauzito? | 1. Ndio 2. Hapana |
| **10.** | Kama ndio, ni aina gani ya kazi ulikuwa ukifanya? | 1. Mama wa nyumbani 2. Muhudumu wa afya 3. Walinzi, polisi 4. Usafi wa barabara 5. Mama ntilie 6. Wafanyakazi wa saluni za kike 7. Wafanyakazi wa ofisini 8. Walimu 9. Wakulima 10. Kilimo cha samaki 11. Viwanda vya nguo 12. Biashara ndogondogo   Nyinginezo taja………………… |
| 11. | Una muda gani unafanya kazi yako ya sasa? | miaka…………………………… |
| 12. | Kwa wiki unafanya kazi siku ngapi? | ………………………… |
| 13. | Je, unafanya kazi kwa shifti (mabadiliko)? | 1. Ndio 2. Hapana |
| 14. | Kama ndio, shift hizo ni za usiku? | 1. Ndio 2. Hapana |
| 15. | Kazi zako ni zipi hasa? | ……………………….. |
| 16. | Je, unapimaje uzito wa kazi yako? | 1. Nyepesi 2. Uzito wa kawaida 3. Kazi nzito 4. Kazi nzito sana |
| **17** | Je, unashughulika na kazi hiyohiyo kila siku? | 1. Ndio 2. Hapana |
| **18.** | Je, kazi inatofautiana siku kwa siku? | 1. Ndio 2. Hapana |
| **19** | Je, mnabadilishana kazi na wafanyakazi wenzako? | 1. Ndio 2. Hapana |
| **20.** | Je kazi yako ni ya kufanya kwa kurudiarudia mara nyingi ndani ya dakika moja? | 1. Ndio 2. Hapana |
| **21** | Una mapumziko mangapi kwa siku za kazi za kawaida? | ………………………………. |
| **22** | Ukihesabu mapumziko yote, huwa una dakika ngapi za kupumzika? | …………………………………. |
| **23** | Je, mapumziko yako uliyonayo yanatosha? | 1. Ndio 2. Hapana |

**ASANTE KWA KUJAZA DODOSO**
