# Supplementary material for: Maternal occupation and risk of adverse fetal outcomes in Tanzania: A hospital-based cross-sectional study
Source: PLoS One. 2025 Mar 18;20(3):e0319653. doi: 10.1371/journal.pone.0319653 (PMC11918367; doi:10.1371/journal.pone.0319653)
Supplement: S2 File — (DOCX) [file pone.0319653.s003.docx]

**English version questionnaire for post-delivery women**

**Questionnaire No**_____________

**Ward**  ___________________

**Date of interview**________________

| **SECTION 1: SOCIAL DEMOGRAPHIC INFORMATION OF POST-DELIVERY WOMEN** | | |
| --- | --- | --- |
| **QUESTION** | | **RESPONSE** |
| 1. | How old are you? *(age in years)* | ……… |
| 2 | Marital Status | 1. Single 2. Married 3. Separated 4. Widow |
| 3. | What is your level of education? (*Tick appropriate*) | 1. No formal education 2. Primary education 3. Secondary education 4. Higher education   (specify)…………… |
| 4. | Do you have any history of diabetes? (*Tick appropriate*) | 1.yes  2.No |
| 5. | Do you have any history of hypertension? (*Tick appropriate*) | 1.yes  2.No |

| **SECTION 2: MATERNAL OCCUPATION** | | |
| --- | --- | --- |
| **6.** | Have you been working before pregnant? | 1. Yes 2. No |
| **7.** | Which kind of job | 1. Home wife 2. Healthcare workers 3. Public servants 4. Road cleaners 5. Food vendors 6. Hairdressers and cosmetologists 7. Office works 8. Teachers 9. Agricultural workers 10. Aquaculture 11. Textile 12. Small business 13. Other………………… |
| **8** | Which characteristics among the following does your job involve? (Tick where appropriate) | 1. Prolonged standing 2. Prolonged sitting 3. Prolonged squatting/ kneeing 4. Moving loads (more than   5Kg)   1. Moving heavy loads (more |

|  |  | than 20Kg)   1. Jobs that require extension of arms/hands 2. Working with vibrating tools 3. Working in a noisy environment 4. Working in a confortable pustules 5. Working in the same position for long periods 6. Doing repetitive tasks many times for one minute 7. Working with chemicals in agriculture 8. Handling chemicals for pests at home |
| --- | --- | --- |
| **9.** | Did you continue working after pregnancy? | 1. Yes 2. No |
| **10.** | Which kind of job? | 1. Home wife 2. Healthcare workers 3. Public servants 4. Road cleaners 5. Food vendors 6. Hairdressers and cosmetologists 7. Office works 8. Teachers 9. Agricultural workers |

|  |  | 1. Aquaculture 2. Textile 3. Small business 4. Other………………… |
| --- | --- | --- |
| 11. | How many years have you been carrying your present work? | …………………………… |
| 12. | How many days per week were you going to work? | ………………………… |
| 13. | Are you working on shifts? | 1. Yes 2. No |
| 14. | Does the shifts include night shift? (*Tick appropriate*) | 1. Yes 2. No |
| 15. | What are your job tasks? | ……………………….. |
| 16. | How do you rank the weight of your tasks (*Tick appropriate*) | 1. Light 2. Normal 3. Heavy 4. Very heavy |
| **17** | Have been carrying out the same activities every day? | 1. Yes 2. No |
| **18.** | Does the work vary from day to day? (Tick appropriate) | 1. Yes 2. No |
| **19** | Does it rotate within your colleagues? | 1. Yes 2. No |
| **20.** | Does you work involve many repetitive tasks many times a minute? (*Tick appropriate*) | 1. Yes 2. No |
| **21** | How many breaks do you have during a normal working day? | ………………………………. |
| **22** | Counting all breaks, how many minutes resting time do you normally have? | …………………………………. |
| **23** | Are your normal breaks sufficient? | 1. Yes 2. No |

**THANK YOU FOR ANSWERING THE QUESTIONNAIRE**
