# Supplementary material for: Maternal occupation and risk of adverse fetal outcomes in Tanzania: A hospital-based cross-sectional study
Source: PLoS One. 2025 Mar 18;20(3):e0319653. doi: 10.1371/journal.pone.0319653 (PMC11918367; doi:10.1371/journal.pone.0319653)
Supplement: S3 File — (DOCX) [file pone.0319653.s004.docx]

**Observation checklist NUMBER OF PATIENT FILE______________**

| **GENERAL INFORMATION** |  |  |
| --- | --- | --- |
| **Parity……………….** |  |  |
| **Gravidity……………………………** |  |  |
| **ANC visits…………………………….** |  |  |
| **MATERNAL COMPLICATION** | **YES** | **NO** |
| 1. Eclampsia |  |  |
| 2. Preeclampsia |  |  |
| 3. Gestational diabetes |  |  |
| 4. Gestational Hypertension |  |  |
| 5. antepartum hemorrhage (APH) |  |  |
| 6. uterine rupture |  |  |
| 7. postpartum hemorrhage |  |  |
| **NEONATAL OUTCOME** |  |  |
| 1. Low Apgar score (AS<7 at 5minute) |  |  |
| 2. Still birth |  |  |
| 3. Low birth weight |  |  |
| 4. Small for gestational age |  |  |
| 5. Early neonatal death |  |  |
