# Supplementary material for: Maternal occupation and risk of adverse fetal outcomes in Tanzania: A hospital-based cross-sectional study
Source: PLoS One. 2025 Mar 18;20(3):e0319653. doi: 10.1371/journal.pone.0319653 (PMC11918367; doi:10.1371/journal.pone.0319653)
Supplement: Attachment — Submitted filename: Occupation and fetal outcome.pdf [file pone.0319653.s005.pdf]

PLOS ONE

Maternal occupation and risk of adverse fetal outcomes in Tanzania: a Hospital- based cross-sectional study

--Manuscript Draft--

**Manuscript Number:**	PONE-D-24-38857
**Article Type:**	Research Article
**Full Title:**	Maternal occupation and risk of adverse fetal outcomes in Tanzania: a Hospital- based cross-sectional study
**Short Title:**	Maternal occupation and risk of adverse fetal outcomes in Tanzania
**Corresponding Author:**	Baldwina Tita Olirk, Masters of Science in Environmental and Occupation<br>Muhimbili National Hospital<br>Dar es Salaam, TANZANIA, UNITED REPUBLIC OF
**Keywords:**	Occupation; Tanzania; post-delivery women; adverse fetal outcome
**Abstract:**	Background<br>Women constitute a large proportion of the workforce in today's world. This workforce consists of women of reproductive age. Hazardous working environment conditions for these women are a threat to their health as well as that of their unborn babies. We assessed whether maternal occupation during pregnancy is associated with adverse fetal outcomes.<br>Methods<br>A cross-sectional study was conducted among 400 self-referred post-delivery women at a referral Hospital in Tanzania. Information on socio-demographic characteristics and maternal occupational characteristics was assessed through the use of a pre-tested questionnaire. Questions on physical demanding work and prolonged standing were obtained from the standardized Musculoskeletal Questionnaire. To assess occupational exposure to chemicals, job titles and task descriptions were linked to a job-exposure-matrix, an expert judgment on exposure to chemicals at the workplace. Information relating to obstetric characteristics and pregnancy outcomes was obtained from the medical files and clinic cards. Data was analyzed by using Statistical Package for Social Sciences (SPSS) version 23. Odds ratios > 1 was considered risk while Odds ratios < 1 was considered protective and P value <0.05 was considered significant.<br>Results:<br>The mean age was 28.0 ± 6.3. Out of 400 post-delivery women studies, 174 (43.5%) were engaged in various occupations. Agriculture (22.4%) was the most prevalent occupation followed by tailoring (19.0%). Relative to the referent group of other occupations, agriculture workers, had higher adjusted odds ratios of congenital malformation (AOR= 6.3, 95% CI; 1.4-28.8) preterm babies (AOR= 3.9, 95% CI; 1.1-15.8), low birth weight (AOR= 4.1, 95% CI; 1.2-16.5) and low Apgar score (AOR=7.4, 95% CI; 2.0-30.0). Food vendors: low birth weight (AOR=5.9, 95% CI; 1.1-30.7) and low Apgar score (AOR=14.1, 95% CI; 2.7-25.1)<br>Conclusion<br>Understanding occupational characteristics and their relation to adverse fetal outcomes is important to formulate appropriate strategies to promote and protect maternal and infant health at work.
**Order of Authors:**	Baldwina Tita Olirk, Masters of Science in Environmental and Occupation
	Aiwerasia Vera Ngowi, PhD
	Furaha August, PhD
	Ezra Jonathan Mrema, PhD
	Jovine Bachwenkizi, PhD
	Simon Henry Mamuya, PhD
**Additional Information:**	
**Question**	**Response**

Powered by Editorial Manager® and ProduXion Manager® from Aries Systems Corporation

**Financial Disclosure**<br><br>Enter a financial disclosure statement that describes the sources of funding for the work included in this submission. Review the submission guidelines for detailed requirements. View published research articles from *PLOS ONE* for specific examples.<br><br>This statement is required for submission and **will appear in the published article** if the submission is accepted. Please make sure it is accurate.<br><br>**Funded studies**<br>Enter a statement with the following details:<br>• Initials of the authors who received each award<br>• Grant numbers awarded to each author<br>• The full name of each funder<br>• URL of each funder website<br>• Did the sponsors or funders play any role in the study design, data collection and analysis, decision to publish, or preparation of the manuscript?<br><br>Did you receive funding for this work?	Yes
Please add funding details.<br>as follow-up to "**Financial Disclosure**<br><br>Enter a financial disclosure statement that describes the sources of funding for the work included in this submission. Review the submission guidelines for detailed requirements. View published research articles from *PLOS ONE* for specific examples.<br><br>This statement is required for submission and **will appear in the published article** if the submission is accepted. Please make sure it is accurate.	Funding was provided by SIDA project from Muhimbili University of Health and Allied Sciences

**Funded studies**<br>Enter a statement with the following details:<br>• Initials of the authors who received each award<br>• Grant numbers awarded to each author<br>• The full name of each funder<br>• URL of each funder website<br>• Did the sponsors or funders play any role in the study design, data collection and analysis, decision to publish, or preparation of the manuscript?<br><br>Did you receive funding for this work?"	
Please select the country of your main research funder (please select carefully as in some cases this is used in fee calculation).<br>as follow-up to "**Financial Disclosure**<br><br>Enter a financial disclosure statement that describes the sources of funding for the work included in this submission. Review the submission guidelines for detailed requirements. View published research articles from *PLOS ONE* for specific examples.<br><br>This statement is required for submission and **will appear in the published article** if the submission is accepted. Please make sure it is accurate.<br><br>**Funded studies**<br>Enter a statement with the following details:<br>• Initials of the authors who received each award<br>• Grant numbers awarded to each author<br>• The full name of each funder<br>• URL of each funder website<br>• Did the sponsors or funders play any role in the study design, data collection and analysis, decision to publish, or preparation of the manuscript?<br><br>Did you receive funding for this work?"	TANZANIA, UNITED REPUBLIC OF - TZ
**Competing Interests**	The authors have declared that no competing interests exist.

Use the instructions below to enter a competing interest statement for this submission. On behalf of all authors, disclose any competing interests that could be perceived to bias this work—acknowledging all financial support and any other relevant financial or non-financial competing interests.<br><br>This statement is required for submission and **will appear in the published article** if the submission is accepted. Please make sure it is accurate and that any funding sources listed in your Funding Information later in the submission form are also declared in your Financial Disclosure statement.<br><br>View published research articles from *PLOS ONE* for specific examples.<br><br>**NO authors have competing interests**<br><br>Enter: *The authors have declared that no competing interests exist.*<br><br>**Authors with competing interests**<br><br>Enter competing interest details beginning with this statement:<br><br>*I have read the journal's policy and the authors of this manuscript have the following competing interests: [insert competing interests here]*<br><br>* typeset	
**Ethics Statement**<br><br>Enter an ethics statement for this submission. This statement is required if the study involved:<br><br>• Human participants<br>• Human specimens or tissue<br>• Vertebrate animals or cephalopods<br>• Vertebrate embryos or tissues	All methods were carried out in accordance with Declaration of Helsink guidelines and regulations. Ethics approval was obtained from ethics committee of Muhimbili University of Health and Allied Sciences with reference number DA.282/298/01.C/938. The permission to conduct the study at the referral hospital was obtained from the Executive Director (ED). Participants 18 years and above were given written informed consent forms to consent to participate in the study while parental/guardian consent was obtained for participants less than 18 years. Although we had 14 participants who had no formal education, they could read and write. The study participants who were found to have adverse fetal outcome were given appropriate care.

Powered by Editorial Manager® and ProduXion Manager® from Aries Systems Corporation

• Field research<br><br>Write "N/A" if the submission does not require an ethics statement.<br><br>General guidance is provided below. Consult the submission guidelines for detailed instructions. **Make sure that all information entered here is included in the Methods section of the manuscript.**	

**Format for specific study types**<br><br>**Human Subject Research (involving human participants and/or tissue)**<br>• Give the name of the institutional review board or ethics committee that approved the study<br>• Include the approval number and/or a statement indicating approval of this research<br>• Indicate the form of consent obtained (written/oral) or the reason that consent was not obtained (e.g. the data were analyzed anonymously)<br><br>**Animal Research (involving vertebrate animals, embryos or tissues)**<br>• Provide the name of the Institutional Animal Care and Use Committee (IACUC) or other relevant ethics board that reviewed the study protocol, and indicate whether they approved this research or granted a formal waiver of ethical approval<br>• Include an approval number if one was obtained<br>• If the study involved *non-human primates*, add *additional details* about animal welfare and steps taken to ameliorate suffering<br>• If anesthesia, euthanasia, or any kind of animal sacrifice is part of the study, include briefly which substances and/or methods were applied<br><br>**Field Research**<br><br>Include the following details if this study involves the collection of plant, animal, or other materials from a natural setting:<br>• Field permit number<br>• Name of the institution or relevant body that granted permission	
**Data Availability**<br><br>Authors are required to make all data underlying the findings described fully available, without restriction, and from the time of publication. PLOS allows rare exceptions to address legal and ethical concerns. See the PLOS Data Policy and FAQ for detailed information.	Yes - all data are fully available without restriction

<table>
<tr><td>A Data Availability Statement describing where the data can be found is required at submission. Your answers to this question constitute the Data Availability Statement and will be published in the article, if accepted.

Important: Stating ‘data available on request from the author’ is not sufficient. If your data are only available upon request, select ‘No’ for the first question and explain your exceptional situation in the text box.

Do the authors confirm that all data underlying the findings described in their manuscript are fully available without restriction?</td><td></td></tr>
<tr><td>Describe where the data may be found in full sentences. If you are copying our sample text, replace any instances of XXX with the appropriate details.

• If the data are held or will be held in a public repository, include URLs, accession numbers or DOIs. If this information will only be available after acceptance, indicate this by ticking the box below. For example: All XXX files are available from the XXX database (accession number(s) XXX, XXX.).
• If the data are all contained within the manuscript and/or Supporting Information files, enter the following: All relevant data are within the manuscript and its Supporting Information files.
• If neither of these applies but you are able to provide details of access elsewhere, with or without limitations, please do so. For example:

Data cannot be shared publicly because of [XXX]. Data are available from the XXX Institutional Data Access / Ethics Committee (contact via XXX) for researchers who meet the criteria for access to confidential data.

The data underlying the results presented in the study are available from (include the name of the third party</td><td>All relevant data are within the manuscript and its Supporting Information files.</td></tr>
</table>

and contact information or URL).<br>• This text is appropriate if the data are owned by a third party and authors do not have permission to share the data.<br><br>* typeset	
Additional data availability information:	Tick here if your circumstances are not covered by the questions above and you need the journal’s help to make your data available.

Manuscript Click here to access/download;Manuscript;Manuscript .doc

Maternal occupation and risk of adverse fetal outcomes in Tanzania: a Hospital- based cross-sectional study

Baldwina Tita Olirk[1]*, Aiwerasia Vera Ngowi,[2] Furaha August,[3] Ezra Jonathan Mrema,[2] Jovine Bachwenkizi,[2] Simon Henry Mamuya[2]

1. Department of Occupational Health and Safety, Muhimbili National Hospital, P.O. Box 65000, Dar es Salaam, Tanzania.
2. Department of Environmental and Occupational Health, Muhimbili University of Health and Allied Sciences, P.O. Box 65015, 9 United Nations Road, Upanga West, Dar es Salaam, Tanzania.
3. Department of Obstetrics and gynecology, School of medicine, Muhimbili University of Health and Allied Sciences, P.O. Box 65001, 9 United Nations Road, Upanga West, Dar es Salaam, Tanzania.

***Correspondence author:** Baldwina Tita Olirk, P.O. Box 65000, Dar es Salaam, Tanzania. Mob: +255766477824; e-mail: baldwina.olirk@mnh.or.tz

Abstract

Background

Women constitute a large proportion of the workforce in today's world. This workforce consists of women of reproductive age. Hazardous working environment conditions for these women are a threat to their health as well as that of their unborn babies. We assessed whether maternal occupation during pregnancy is associated with adverse fetal outcomes.

Methods

A cross-sectional study was conducted among 400 self-referred post-delivery women at a referral Hospital in Tanzania. Information on socio-demographic characteristics and maternal occupational characteristics was assessed through the use of a pre-tested questionnaire. Questions on physical demanding work and prolonged standing were obtained from the standardized Musculoskeletal Questionnaire. To assess occupational exposure to chemicals, job titles and task descriptions were linked to a job-exposure-matrix, an expert judgment on exposure to chemicals at the workplace. Information relating to obstetric characteristics and pregnancy outcomes was obtained from the medical files and clinic cards. Data was analyzed by using Statistical Package for Social Sciences (SPSS) version 23. Odds ratios > 1 was considered risk while Odds ratios < 1 was considered protective and P value <0.05 was considered significant.

Results:

The mean age was 28.0 ± 6.3. Out of 400 post-delivery women studies, 174 (43.5%) were engaged in various occupations. Agriculture (22.4%) was the most prevalent occupation followed by tailoring (19.0%). Relative to the referent group of other occupations, agriculture workers, had

higher adjusted odds ratios of congenital malformation (AOR= 6.3, 95% CI; 1.4-28.8) preterm babies (AOR= 3.9, 95% CI; 1.1-15.8), low birth weight (AOR= 4.1, 95% CI; 1.2-16.5) and low Apgar score (AOR=7.4, 95% CI; 2.0-30.0). Food vendors: low birth weight (AOR=5.9, 95% CI; 1.1-30.7) and low Apgar score (AOR=14.1, 95% CI; 2.7-25.1)

Conclusion

Understanding occupational characteristics and their relation to adverse fetal outcomes is important to formulate appropriate strategies to promote and protect maternal and infant health at work.

Key words: Occupation, Tanzania, post-delivery women, adverse fetal outcome

Introduction

Adverse fetal outcome has been a worldwide threat and remains to be a major cause of perinatal morbidity and mortality (1–3). Globally, it was reported that 2·42 million neonates died in 2019 with the majority of death occurring in Sub-Saharan Africa and South Asia (4). During an assessment of under-five mortality rate, it was seen that the rate in Sub-Saharan Africa and South Asia increased from 73% (7·07 million deaths) in 2000 to 80% (4·04 million deaths) in 2019 (4). Risk factors for adverse fetal outcomes include maternal age, body mass index, obstetric factors and pregnancy-related syndromes including hypertensive disorders in pregnancy and other medical co-morbidities like HIV, Malaria, Anemia and sickle cell disease (5–7).

Women constitute almost half of the workforce globally, majority work during their reproductive age (8). In England, the USA and Iran, 70%, 59% and 13% of women are employed respectively (9). In Tanzania, according to the World Bank collection of development indicators report of 2020,

female labor force, accounts for 48.13% (10). This group include pregnant women and women of reproductive age. A large portion of these women work in informal sectors and hazardous occupational conditions including long periods of standing, long periods of walking, lifting or carrying heavy weights, night shifts, long working hours, exposure to chemicals such as pesticides, phthalates, organic solvents, alkylphenolic compounds, heavy metals and other chemicals (11). The exposure to such hazardous occupational conditions may affect negatively the fetal outcome. An analysis of 13 European birth cohorts that evaluated the influence of maternal occupation on birth weight, and length of gestation revealed higher odds of low birth weight (LBW) babies and longer length of gestation for the women who worked in construction industry (8). Likewise, it was observed that women who worked in food industry had higher odds of preterm delivery and reduced length of gestation.

Additional studies on occupational exposures such as chemicals and noise has been related with reproductive defects in women and effects in pregnancy (11,12). Numerous studies have acknowledged that occupational exposure to chemicals and toxins to women working in different occupations such as pesticides from agriculture, chemicals from salons, health centers and heavy metals can lead to teratogenic effect to their unborn babies (11,13). A study that was done in Florida among female agricultural workers while pregnant revealed that, female working in agriculture sector reported more health symptoms during their pregnancy than female workers in a control group (14). In addition in studies in chemical exposure, a study among cosmetologists and manicurists in California revealed that women who work in these industries during pregnancy are at higher risks for small for gestational age (SGA) compared to other working women and general population (15).

Maternal employment during pregnancy in relation to fetal outcome has been rarely studied and most of them have generated contradictory findings (16). More studies are focused on Prevention of Mother to Child Transmission (PMTCT) program due to surge HIV transmissions among young adults. On the other hand, it is still not clear on how women in Tanzania should be handled during the maternal period. This study therefore will assess whether maternal occupation during pregnancy is associated with adverse fetal outcomes. The results of the study will broaden knowledge about health of pregnant women and their fetus. The findings will also be useful to researchers for conducting future research, and for policy makers to review the policies to include issues that address how women should be handled during the maternal period.

Materials and methods

Study Design

This analytical cross-sectional study was conducted to determine whether maternal occupation during pregnancy is associated with adverse fetal outcomes.

Setting

The Study was conducted at Muhimbili National Hospital (MNH). MNH is the largest referral hospital in Tanzania and a teaching hospital for Muhimbili University of Health and Allied Sciences. The Hospital is located in Dar-es-Salaam city with a population of 5.4million (Tanzania Demographic Census 2022). It serves populations of different levels of education and socioeconomic status. It also attends referred patients from different regions and so caters to a large number of wide ranges of patients including those with diverse occupations and pregnancy outcomes. MNH also harbors many specialists who are capable of making correct diagnosis. It is the largest referral hospital in the United Republic of Tanzania with 1,500 bed facility, attending 1,000 to 1,200 outpatients per day, admitting 1,000 to 1,200 inpatients per

week. The hospital receives maternal cases mostly from the regional hospitals and very few from outside the country. The hospital has 2 maternity blocks in which women with pregnancies or having delivered pregnancies of 28 weeks or more are admitted. There are seven wards, four wards are reserved for admission of women with antenatal and postnatal complications and women with sick children. The number of deliveries per day is between 20 and 40. Also, there are two gynecological wards, each with 32 beds. The gynecological wards admit women with pregnancies of less than 28 weeks of gestation who have developed complications together with non-pregnant women with gynecological conditions. Emergency operations are conducted either in the Emergency medicine operation theatre or a gynecological operating room at the main theatre. The site was purposively selected with consideration of high number of deliveries.

Study subjects and sampling methodology

The study was conducted from April to June 2022. The method used for sample size estimation was probability sample size estimation developed by Bhopal (17). We were able to obtain a sample size of 400 with a response rate of 80%. Purposive sampling was conducted including working women (paid labour and self-employed) and non-working women within 72 h of delivery. An inclusion criterion was singleton pregnancy leading to live birth. The exclusion criteria were maternal cormobities like cardiac, renal disease, sickle cell and HIV. To account for the design effect, the sample size was multiplied by 1.5.

Data collection procedure and tools

Exposure

A combination of occupation (job title) evidenced to be performed by women in Tanzania was considered for all jobs held by mothers. Jobs held from three months before pregnancy to the end of the third pregnancy month were considered exposures**.** A team of two occupational experts collected information on mothers' employment status and occupational history. Up to eight jobs held during pregnancy and 3 months before pregnancy were recorded and coded for each woman. However, only jobs held during the critical period of fetal development, defined as 1 month prior to pregnancy through the end of the third pregnancy month, were included in these analyses. Questions on physical demanding work, manually handling loads of 25 kg and above, prolonged standing, night shifts, and working hours were obtained from the standardized Musculoskeletal Questionnaire. Information on work schedules included the nature of shift (either fixed verses rotating shift) and night shift. Information on working hours was calculated by number of working hours per day in the category of <8 hours and >8 hours. Mothers were also asked to rank the weight of their tasks into (light, normal, heavy, very heavy). Mothers were also asked to estimate the length of experience (in years) for the current job. To assess occupational exposure to chemicals, job titles and task descriptions were linked to a job-exposure-matrix (JEM), an expert judgment on exposure to chemicals at the workplace.

A face-to-face interview with delivering mothers was conducted using a structured questionnaire. Together with the collection of occupation related information described above, the questionnaire also gathered information on socio-demographic information (such as age, educational status, marital status). The questionnaire was written in English and translated into Swahili.

Outcome Variable

The outcome variable was adverse fetal outcome. These included low birth weight, early neonatal death, low APGAR scores, prematurity, stillbirths and physical congenital anomalies. Low birth weight was defined as birth weight of less than 2500grams, prematurity as neonates born at less than 37 weeks gestational age, still birth as the baby with no sign of life at or after 28 weeks of gestational age, low Apgar score as a score below 7. This information was obtained from the medical records and transferred to a structured checklist with the help of trained midwives. Addition to foetal information obstetric data was also collected including gravidity, parity, gestational age at booking and other maternal complication such as eclampsia, preeclampsia, gestational diabetes, chronic hypertension, APH and uterine rupture.

Data Analysis

The dependent variable, adverse fetal outcome (i.e. low birth weight, early neonatal death, low APGAR scores, prematurity, stillbirths and physical congenital anomalies), were measured as categorical variables where 1 is the presence of any adverse fetal outcome and 2 was the absence of adverse fetal outcome. The prevalence of adverse fetal outcome was calculated by the formula developed by Bhopal (17).

Statistical analysis

The data was analyzed by using IBM Statistical Package for Social Sciences (SPSS) for Windows version 23 (IBM Corp., Armonk, New York, USA) applying both descriptive and inferential statistical approaches. Inferential statistical approaches applied were chi-square test and logistic regression where p-value less than 0.05 was considered statistically significant. Odds ratios > 1 was considered as risk while Odds ratios < 1 was considered protective. Chi-square test was used to show the relationship between adverse fetal outcome and independent variables. Logistic regression

was used to determine factors which affected the outcome variable independently while correcting for confounders (age, BMI, obstetric factors).

Ethics approval and consent to participate

All methods were carried out in accordance with Declaration of Helsink guidelines and regulations. Ethics approval was obtained from ethics committee of Muhimbili University of Health and Allied Sciences with reference number DA.282/298/01.C/938. The permission to conduct the study at the referral hospital was obtained from the Executive Director (ED). Participants 18 years and above were given written informed consent forms to consent to participate in the study while parental/guardian consent was obtained for participants less than 18 years. Although we had 14 participants who had no formal education, they could read and write. The study participants who were found to have adverse fetal outcome were given appropriate care.

Results

Background characteristics of study participants

We contacted 400 post-delivery women resulting in a participation rate of 70%. The mean age, gravidity, parity, and ANC visits of the study population were 28.0± 6.3, 3± 2, 2± 1, and 4.5± 1.9, respectively. Almost half of study participants had primary education (47.5%) and majority being married (74.2%). The rest of the characteristics are shown in **Table 1**

Table1: General characteristics of study participants (N = 400)

Character	Frequency (n)	Percentage (%)
**Age (years)**		
15-19	35	8.8%
20-24	95	23.8%

25-29	113	28.2%
>29	157	39.2%
**Education level**		
No formal education	14	3.5%
Primary education	109	47.5%
Secondary education	133	33.3%
Higher education	63	15.8%
**Marital status**		
Single	103	25.8%
Married	297	74.2%
**History of diabetes**		
Yes	18	4.5%
No	382	95.5%
**History of hypertension**		
Yes	36	9.0%
No	364	91.0%
**Alcohol use during Pregnancy**		
Yes	22	5.5%
No	378	94.5%

Prevalence of adverse fetal outcomes

Among 174 (43.5%) working women, 153 (38.25%) had at least one adverse fetal outcome. The commonest adverse outcomes in working women were low birth weight (27.1%) followed by

preterm babies (24.5%). **Table 2** shows distribution of risk for adverse fetal outcomes between women who were working and those who were not working during pregnancy.

Table 2: Distribution of adverse fetal outcome among working and non-working women

**Neonatal Outcome**	**Category**		**P value**
	**Working women**	**Non-working women**	
**Presence of any adverse outcome**			
Yes	153 (87.9%)	162 (71.7%)	<0.0001
No	21 (12.1%)	64 (28.3%)	
**Low Apgar score at 5 minutes**			
Yes	71 (40.8%)	45 (19.9%)	<0.0001
No	103 (59.2%)	181 (80.1%)	
**Still birth**			
Yes	15 (8.6%)	7 (3.1%)	0.016
No	159 (91.4%)	219 (96.9%)	
**Low birth weight**			
Yes	108 (62.1%)	66 (37.9%)	<0.0001
No	83 (36.7%)	209 (52.3%)	
**Preterm babies**			
Yes	98 (56.3%)	72 (31.9%)	<0.0001
No	76 (43.7%)	154 (68.1%)	
**Early neonatal death**			
Yes	5 (2.9%)	12 (5.3%)	0.231
No	169 (97.1%)	214 (94.7%)	

Congenital anomaly			
Yes	55 (31.6%)	43 (19.0%)	0.004
No	119 (68.4%)	183 (81.0%)	

Distribution of risk factors

Occupational Status

In our study, post-delivery women self-reported to engage in various jobs during pregnancy (**Figure 1**). Out of 400 post-delivery women studied, 174 (43.5%) were engaged in various occupation. The most prevalent occupation was agriculture (22.4%) followed by tailoring (19.0%).

Figure 1: Job titles for study participants

Relationships between adverse fetal outcome and maternal occupation

Working in agriculture sector during pregnant had higher adjusted odds of congenital malformation (AOR=6.3, 95% CI; 1.4-28.8) preterm babies (AOR=3.9, 95% CI; 1.1-15.8), low birth weight (AOR=4.1, 95% CI; 1.2-16.5) and low Apgar score at 5minutes (AOR=7.4, 95% CI; 2.0-30.0). The rest of the variables are shown in **Table 3**.

Table 3: Association of maternal occupation and fetal outcomes

Congenital malformation	p value	COR (95% CI)	P value	AOR (95% CI)
**Occupation type/characteristics**				

Food vendors	0.090	0.2 (0.05-1.2)	0.075	0.2 (0.04-1.1)
Working in Agriculture	0.001	5.3 (1.9-14.7)	0.002	5.1 (1.8-14.4)
**Preterm babies**				
**Occupation type/characteristics**				
Working in Agriculture	0.083	2.5 (0.9-6.8)	0.049	3.9 (1.1-15.8)
**Low birth weight**				
**Occupation type/characteristics**				
Food vendors	0.022	4.0 (1.2-13.2)	0.012	4.8 (1.4-16.3)
Working in Agriculture	0.026	3.1 (1.2-8.3)	0.014	3.6 (1.3-9.6)
**Low Apgar score at 5 minutes**				
**Occupation type/characteristics**				
Working in Agriculture	0.015	3.5 (1.3-9.3)	<0.0001	13.7 (8.7-31.7)
Food vendors	>0.001	13.3 (3.6-46.8)	0.013	3.5 (1.3-9.6)
**Controlled for gestation hypertension, gestation diabetes, pre eclampsia and eclampsia**				

Discussion

In this study, we assessed maternal occupations associated with adverse fetal outcome among post-delivery women in Tanzania. The commonest type of occupation was agriculture, tailoring and hairdressers. This finding is not surprising as majority of women in Tanzania are engaged mainly in informal sectors like Agriculture and Hairdressing (18,19).

The type of occupation positively associated with the largest number of adverse fetal outcome was agriculture. Working in agricultural sector was positively associated with congenital malformation, low birth weight, low Apgar score and preterm babies. The higher odds of adverse fetal outcomes

among agricultural female workers is related to pesticide exposure (20,21). Women agricultural workers constitute a key occupational group of workers who experience pesticide exposure at childbearing age (18). They experience indirect exposure through harvesting, planting, and soil preparation, re-entry activities such as gathering and bunching flowers, clean-up activities like washing contaminated clothes of their husbands and handling/reusing pesticides empty containers. Epidemiologic and animal studies have acknowledged that occupational exposure to chemicals and toxins to women working in different occupations such as pesticides from agriculture, can lead to adverse effect to their unborn babies (22,23)

These effects have also been pronounced elsewhere. For example, a case–control study by Addissie et al. examined whether maternal exposure to pesticides increased the risk of birth defects (21). A high prevalence of congenital malformation at birth was revealed among cases than controls. Harley et al. conducted a study to evaluate whether maternal pesticide exposure had effect on the new born (24). The study showed decreased foetal growth and length of gestation in new-borns whose mothers were exposed to pesticides during pregnancy. Ling et al in 2018 studied prenatal exposure to pesticides in connection with preterm birth and low birth weight and found a strong association of preterm birth with pesticide exposure (20). In addition, ecological and cross-sectional studies have conveyed positive associations between adverse foetal outcome (particularly preterm birth and low birth weight) and prenatal pesticide exposure (25,26). Small biomarker-based studies with measured pesticides and their metabolites in maternal blood, urine, or umbilical cord blood have reported positive associations with the adverse foetal outcome particularly preterm birth and low birth weight (27,28).

Mothers who were food vendors were at higher risk of delivering newborns with low birth weight and low Apgar score. This finding is supported by other studies. Amegah and colleagues found an association of low birth weight in street food vendors (29). This association might be due to automobile air pollution and wind-blown dust emission from the roads (30). In Tanzania high levels of particulate matter ranging from 11-20 μg/m3 have been reported since 2005 (30). Studies in well resource countries have documented pregnancy outcome and air pollution by assessing proximity of the residents to the roads. For instance, a study in Japan by Yorifuji et al reported residents within ≤50 m to be 1.5 times of getting newborn with low birth weight (31). According to a thorough analysis of the data on ambient air pollution by Stieb et al, it was revealed that there was a 11.4 g decrease in birth weight per 1 ppm CO, 28.1 g decrease per 20 ppb NO_2 and 16.8 g per 20 g/m^3 PM10 (32). A study involving fifteen countries in Africa showed significant associations between prenatal exposure to particulate matter and increased odds of LBW (30).

This study had several limitations. The JEM is used to measure occupational exposure at the job level, which may have led to exposure classification errors. Circumstances at the workplace are often unpredictable and can vary within jobs, between workplaces and over time. It's also possible that women avoided specific exposures while working in a position that would typically expose them because they desired to get pregnant or were aware that they were pregnant.

Conclusion

This study contains findings from analyses on a variety of jobs and fetal outcome in order to generate hypotheses. It also provides information that will help direct future research in relation to adverse fetal outcome and workplace hazards. At least one adverse fetal outcome was seen in

38.25% of the working women. The commonest adverse outcomes in working women were low birth weight (27.1%) followed by preterm babies (24.5%). The type of occupation/job positively associated with several adverse fetal outcomes included agriculture and street food vendors. These findings point to the necessity of exercising caution while scheduling job obligations during pregnancy and carefully monitoring the fetus' growth during prenatal appointments. Future studies should build on these findings by assessing the maternal outcome and its relation to their work.

Acknowledgement

The authors are grateful for the financial support provided by SIDA project. We would also like to thank post-delivery mothers who spared their precious time to participate in our study.

Reference

1. Vats K, Paul M. Study of fetal outcome in hypertensive disorders of pregnancy in a tertiary care maternity hospital of Delhi. Int J Reprod Contraception, Obstet Gynecol. 2016;5(11):3773–7.

2. Xiong T, Mu Y, Liang J, Zhu J, Li X, Li J, et al. Hypertensive disorders in pregnancy and stillbirth rates: A facility-based study in China. Bull World Health Organ. 2018;96(8):531–9.

3. Asseffaid NA, Demissie BW. Perinatal outcomes of hypertensive disorders in pregnancy at a referral hospital, Southern Ethiopia. PLoS One. 2019;14(2):1–10.

4. Paulson KR, Kamath AM, Alam T, Bienhoff K, Abady GG, Abbas J, et al. Global, regional, and national progress towards Sustainable Development Goal 3.2 for neonatal and child health: all-cause and cause-specific mortality findings from the Global Burden of Disease Study 2019. Lancet. 2021;398(10303):870–905.

5. GetahunW.T, SolomonA.A., KassieF.Y., KasayeH.K., DenekewH.T. Uterine rupture among mothers admitted for obstetrics care and associated factors in referral hospitals of Amhara regional state, institution-based cross-sectional study, Northern Ethiopia, 2013-2017. PLoS One [Internet]. 2018;13(12):1–14. Available from: http://www.embase.com/search/results?subaction=viewrecord&from=export&id=L625292349%0Ahttp://dx.doi.org/10.1371/journal.pone.0208470

6. Mrema D, Lie RT, Østbye T, Mahande MJ, Daltveit AK. The association between pre pregnancy body mass index and risk of preeclampsia: A registry based study from Tanzania.

BMC Pregnancy Childbirth. 2018;18(1):1–8.

7. Chuwa FS, Mwanamsangu AH, Brown BG, Msuya SE, Senkoro EE, Mnali OP, et al. Maternal and fetal risk factors for stillbirth in Northern Tanzania: A registry-based retrospective cohort study. PLoS One. 2017;12(8):1–10.

8. Casas M, Cordier S, Martínez D, Barros H, Bonde JP, Burdorf A, et al. Maternal occupation during pregnancy, birth weight, and length of gestation: Combined analysis of 13 European birth cohorts. Scand J Work Environ Heal. 2015;41(4):384–96.

9. Khojasteh F, Arbabisarjou A, Boryri T, Safarzadeh A, Pourkahkhaei M. The Relationship between Maternal Employment Status and Pregnancy Outcomes. Glob J Health Sci. 2016;8(9):37.

10. Idris I. Mapping women's economic exclusion in Tanzania. 2018.

11. Katz VL. Work and work-related stress in pregnancy. Clin Obstet Gynecol. 2012;55(3):765–73.

12. Mrema EJ, Ngowi A V, Mphil SHDM. Status of Occupational Health and Safety and Related Challenges in Expanding Economy of Tanzania Status of Occupational Health and Safety and Related Challenges in Expanding Economy of Tanzania. Ann Glob Heal [Internet]. 2015;81(4):538–47. Available from: http://dx.doi.org/10.1016/j.aogh.2015.08.021

13. Faruk Yorulmaz PhD M. The Effect of Occupational Risk Factors on Pregnancy and Newborn Infants of Pregnant Midwives and Nurses in Turkey: A Prospective Study. Int J Caring Sci. 2017;10(2):690.

14. Runkle J, Flocks J, Economos J, Tovar-Aguilar JA, McCauley L. Occupational risks and pregnancy and infant health outcomes in florida farmworkers. Int J Environ Res Public Health. 2014;11(8):7820–40.

15. Quach T, Von Behren J, Goldberg D, Layefsky M, Reynolds P. Adverse birth outcomes and maternal complications in licensed cosmetologists and manicurists in California. Int Arch Occup Environ Health. 2015;88(7):823–33.

16. Cai C, Vandermeer B, Khurana R, Nerenberg K, Featherstone R, Sebastianski M, et al. The impact of occupational activities during pregnancy on pregnancy outcomes: a systematic review and metaanalysis. Am J Obstet Gynecol [Internet]. 2020;222(3):224–38. Available from: https://doi.org/10.1016/j.ajog.2019.08.059

17. Bhopal RS. Concepts of Epidemiology : [Internet]. Great Clarendon Street,Oxford: Oxford University Press Inc., New York; 2002. Available from: https://www.skums.ac.ir/dorsapax/userfiles/file/Epidemiology_Concepts_2002.pdf

18. Mrema EJ, Ngowi AV, Kishinhi SS, Mamuya SH. Pesticide Exposure and Health Problems Among Female Horticulture Workers in Tanzania. Environ Heal Insights Vol. 2017;11(1):1–13.

19. Tita OB, Mrema EJ, Mamuya S. Ocular symptoms and associated factors among hairdressers in Kinondoni Municipality , Dar es Salaam , Tanzania. East Africa J Appl Heal Monit Eval [Internet]. 2020;1(4):22–7. Available from: file:///C:/Users/baldwina/Downloads/249333-Article Text-596299-1-10-20230613.pdf

20. Ling C, Id ZL, Ehrenstein OS Von, Id JEH. Birth and Term Low Birthweight in Agricultural.

Toxics. 2018;6(41):1–18.

21. Addissie YA, Kruszka P, Troia A, Wong ZC, Everson JL, Kozel BA, et al. Prenatal exposure to pesticides and risk for holoprosencephaly : a case-control study. Environ Heal. 2020;19(65):1–13.

22. Kartin A, Subagio HW, Hadisaputro S, Kartasurya MI, Suhartono S, Budiyono B. Pesticide Exposure and Stunting among Children in Agricultural Areas. Int J Occup Environ Med [Internet]. 2019 Jan 30;10(1):17–29. Available from: http://www.theijoem.com/ijoem/index.php/ijoem/article/view/1428

23. Naksen W, Prapamontol T, Mangklabruks A, Thavornyutikarn P, Srinual N, Panuwet P, et al. Associations of maternal organophosphate pesticide exposure and PON1 activity with birth outcomes in SAWASDEE birth cohort, Thailand. Environ Res. 2016;142(1):288–96.

24. Harley KG, Huen K, Schall RA, Holland NT, Bradman A, Barr DB, et al. Association of Organophosphate Pesticide Exposure and Paraoxonase with Birth Outcome in Mexican-American Women. PLoS ONE |. 2011;6(8).

25. Siqueira MT, Braga C, Cabral-Filho JE, Augusto LGDS, Figueiroa JN, Souza AI. Correlation between pesticide use in agriculture and adverse birth outcomes in Brazil: An ecological study. Bull Environ Contam Toxicol. 2010;84(6):647–51.

26. Rezende Chrisman J, Mattos IE, Koifman RJ, Koifman S, Moraes Mello Boccolini P, Meyer A. Prevalence of very low birthweight, malformation, and low Apgar score among newborns in Brazil according to maternal urban or rural residence at birth. J Obstet Gynaecol Res. 2016;42(5):496–504.

27. Saunders L, Kadhel P, Costet N, Rouget F, Monfort C, Thomé JP, et al. Hypertensive disorders of pregnancy and gestational diabetes mellitus among French Caribbean women chronically exposed to chlordecone. Environ Int [Internet]. 2014;68(1):171–6. Available from: http://dx.doi.org/10.1016/j.envint.2014.03.024

28. Wang P, Tian Y, Wang XJ, Gao Y, Shi R, Wang GQ, et al. Organophosphate pesticide exposure and perinatal outcomes in Shanghai, China. Environ Int [Internet]. 2012;42(1):100–4. Available from: http://dx.doi.org/10.1016/j.envint.2011.04.015

29. Amegah AK, Jaakkola JJK. Work as a street vendor, associated traffic-related air pollution exposures and risk of adverse pregnancy outcomes in Accra, Ghana. Int J Hyg Environ Health [Internet]. 2014;217(2–3):354–62. Available from: http://dx.doi.org/10.1016/j.ijheh.2013.07.010

30. Bachwenkizi J, Liu C, Meng X, Zhang L, Wang W, van Donkelaar A, et al. Maternal exposure to fine particulate matter and preterm birth and low birth weight in Africa. Environ Int [Internet]. 2022;160(December 2021):107053. Available from: https://doi.org/10.1016/j.envint.2021.107053

31. Yorifuji T, Naruse H, Kashima S, Takao S, Murakoshi T, Doi H, et al. Residential proximity to major roads and adverse birth outcomes: A hospital-based study. Environ Heal A Glob Access Sci Source. 2013;12(1):1–11.

32. Stieb DM, Chen L, Eshoul M, Judek S. Ambient air pollution, birth weight and preterm birth: A systematic review and meta-analysis. Environ Res [Internet]. 2012;117:100–11. Available from: http://dx.doi.org/10.1016/j.envres.2012.05.007

Supporting Documents

S1 Fig. This is the Job titles for study participants

S1 Data. The data set used for analysis

S1 File. This is the questionnaire in Kiswahili.

S2 File. This is the questionnaire in English.

S3 File. This is the observation checklist

 Click here to access/download;Figure;S1 Fig. The Job titles for study participants.jpeg

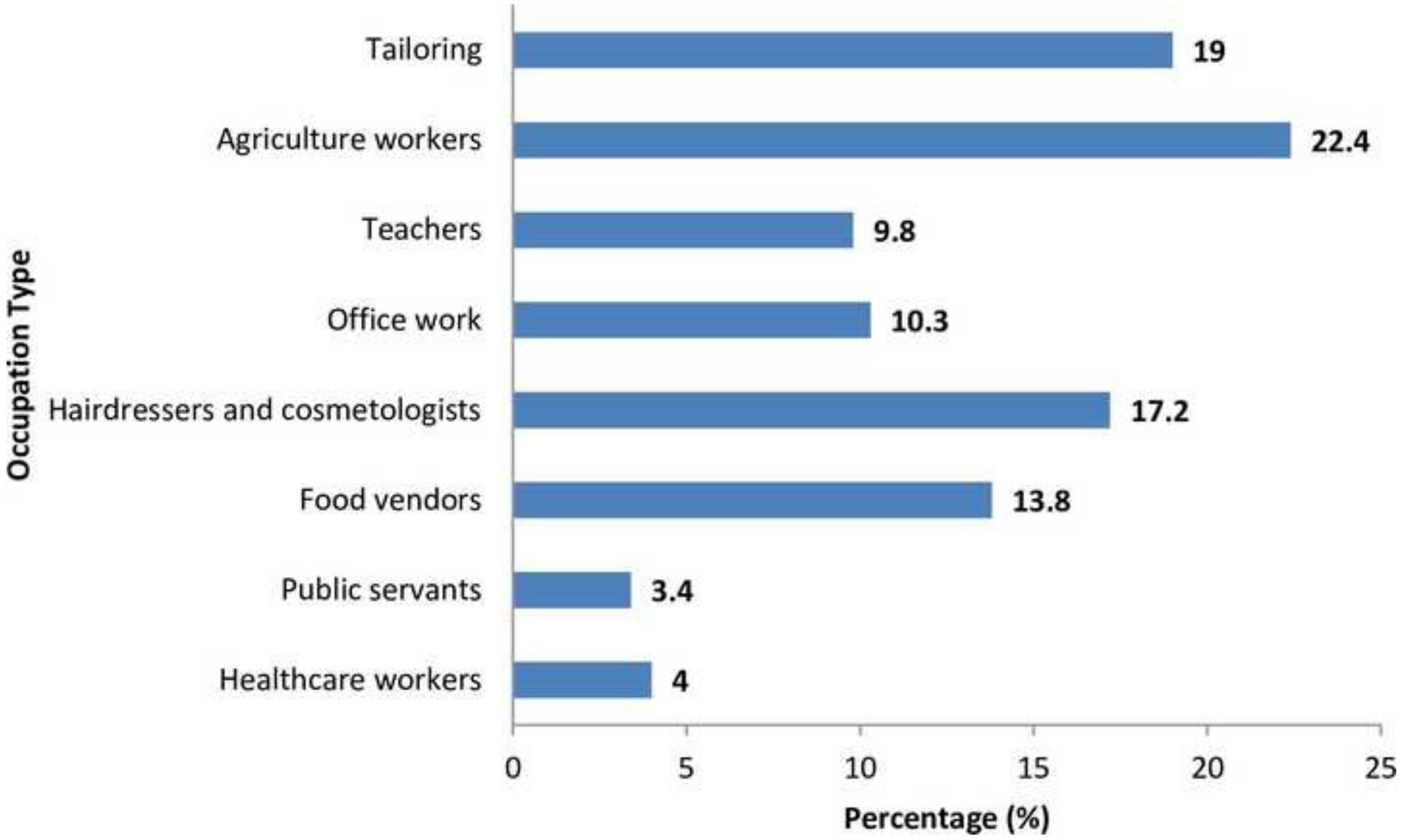

Click here to access/download
Supporting Information
S2 File. The questionnaire in English.docx

Click here to access/download
Supporting Information
S1 File. The questionnaire in Kiswahili.docx

Click here to access/download
Supporting Information
S3 File. The observation checklist.docx

Click here to access/download
Supporting Information
S1 Data. The data set used for analysis.xlsx
